# Temporal distortion may mediate the association between problematic mobile gaming and delay discounting: An experimental study

**DOI:** 10.1556/2006.2025.00092

**Published:** 2025-11-24

**Authors:** Da-I Huang, Ting-Hsi Chou, Chih-Chun Huang, Yun-Hsuan Chang, Chieh-Liang Huang, Mark D. Griffiths, Marc N. Potenza

**Affiliations:** 1 Department of Psychology, Asia University, Taichung, Taiwan; 2 Department of Psychiatry, Taichung Veterans General Hospital, Taichung, Taiwan; 3 Department of Psychiatry, National Cheng Kung University Hospital, Douliou Branch, Yunlin, Taiwan; 4 Department of Psychiatry, College of Medicine, National Cheng Kung University, Tainan, Taiwan; 5 Institute of Gerontology, College of Medicine, National Cheng Kung University, Tainan, Taiwan; 6 Institute of Behavioral Medicine, College of Medicine, National Cheng Kung University, Tainan, Taiwan; 7 Department of Psychology, National Cheng Kung University, Tainan, Taiwan; 8 Department of Neurology, National Cheng Kung University Hospital, Tainan, Taiwan; 9 Institute of Genomics and Bioinformatics, College of Life Sciences, National Chung Hsing University, Taichung, Taiwan; 10 Department of Psychiatry, Tsaotun Psychiatric Center, Ministry of Health and Welfare, Nantou, Taiwan; 11 International Gaming Research Unit, Psychology Department, Nottingham Trent University, Nottingham, UK; 12 Department of Psychiatry and Child Study Center, Yale School of Medicine, New Haven, CT, USA; 13 Connecticut Mental Health Center, New Haven, CT, USA; 14 Connecticut Council on Problem Gambling, Wethersfield, CT, USA; 15 Department of Neuroscience and Wu Tsai Institute, Yale University, New Haven, CT, USA

**Keywords:** problematic mobile gaming, time perception, impulsivity, delay discounting

## Abstract

**Background:**

Impulsivity and delay discounting are considered core components of addiction and are increasingly associated with problematic mobile gaming. However, the mechanisms underlying this association remain unclear. Emerging evidence suggests that altered time perception may contribute to impulsive decision-making in addictive behaviors. Therefore, the present study compared differences in time perception and delay discounting between problematic and non-problematic mobile gamers, and explored the mediating roles of impulsivity and time perception.

**Methods:**

A total of 98 participants were recruited via an online platform and completed a battery of assessments, including the Problematic Mobile Gaming Questionnaire (PMGQ), Barratt Impulsiveness Scale (BIS-11), a time perception task, and a Delay Discounting Task (DDT). Participants were categorized into two groups: problematic mobile gamers (PMGs; *n* = 21) and non-problematic mobile gamers (NPMGs; *n* = 77).

**Results:**

Compared to NPMGs, PMGs showed significantly higher levels of impulsivity, delay discounting (k), and a relative error rate of time perception in 60 s (Rer_60_). A parallel mediation analysis showed that PMGQ score significantly predicted BIS-11 and Rer_60_ (*β* = .44 and .41, *p* < .001). Rer_60_ marginally predicted delay discounting rate (*β* = .18, *p* = .09), whereas the BIS-11 score did not. The total indirect effect was significant (*β* = .01, 95% CI [.0018, .0148]), with Rer_60_ emerging as the primary mediator.

**Conclusion:**

The findings suggest that time perception distortion, rather than impulsivity, mediates the association between problematic mobile gaming and delay discounting.

## Introduction

Mobile gaming has rapidly become a popular form of leisure in Taiwan (where the present study was conducted). According to the Taiwan Internet Report (2023), approximately 31.4% of individuals reported playing mobile games in the past three months. With the widespread availability of smartphones and the advancement of mobile technology, mobile games have become increasingly accessible, contributing to longer playtime and higher gaming intensity on mobile phones (Syvertsen, Ortiz de Gortari, King, & Pallesen, 2022). This growing engagement has raised concerns about problematic mobile gaming behavior, which refers to excessive and compulsive mobile game use that interferes with daily functioning and well-being (Seok & DaCosta, 2018). Problematic mobile gaming is often considered a form of gaming disorder, and its portability, constant connectivity, and immediate access are features that make it particularly risky (Syvertsen et al., 2022).

In a study by Pan, Chiu, and Lin (2019), the prevalence of problematic mobile gaming among Taiwanese students was estimated at 19.7%, significantly higher than the 3.1% prevalence rate of internet gaming disorder (IGD) in Taiwan (Chiu, Pan, & Lin, 2018). Problematic mobile game use (PMGU) has been associated with a range of psychosocial difficulties, including social anxiety (Hong, Chiu, & Huang, 2012; Soni, Upadhyay, & Jain, 2017; Wang, Sheng, & Wang, 2019), depression (Çağan, Ünsal, & Çelik, 2014; Geng, Gu, Wang, & Zhang, 2021; Soni et al., 2017; Thomee, Harenstam, & Hagberg, 2011; Wang et al., 2019), loneliness (Gan, Zhang, Zhang, Wu, & Shao, 2022; Wang et al., 2019) and poor sleep quality (Alahdal, Alsaedi, Garrni, & Alharbi, 2023; Soni et al., 2017; Thomee et al., 2011). Despite these concerns, research has primarily focused on traditional videogames or online gaming, with relatively limited attention paid to the unique impact of mobile gaming (Jang & Ryu, 2016; Wang et al., 2019).

Mobile games offer several advantages over traditional PC games, particularly in terms of convenience and accessibility. Unlike PC games, which typically require a fixed location and extended time commitment, mobile games are designed for short, flexible play sessions that can be started or paused at any moment, making them ideal for filling brief periods of downtime. Many mobile games incorporate psychologically engaging features such as visible progress bars, attainable goals, intermittent rewards, and elements of uncertainty, all of which may contribute to problematic gaming behaviors (Syvertsen et al., 2022; Zendle et al., 2023).

A widely implemented feature in mobile gaming is the “gacha” mechanic, which derives its name from Japanese capsule-toy vending machines (“gachapon”). This system introduces a randomized reward structure whereby players spend in-game currency, often purchased with real money, in exchange for a chance to obtain virtual items or characters of varying rarity. The probabilistic nature of the rewards closely resembles gambling because outcomes are unpredictable and the acquisition of high-value or rare items typically requires repeated attempts. Consequently, gacha systems incentivize prolonged engagement and monetary investment by leveraging intermittent reinforcement schedules, a mechanism known to increase the likelihood of compulsive behavior (Li, Xu, Hao, Xiao, & Liu, 2022). In addition, structural characteristics such as in-game countdown timers, limited-time offers, and visually stimulating reward animations further enhance the psychological appeal of gacha mechanics, contributing to intensified gaming behavior and increased spending among users (Syvertsen et al., 2022). These design elements are known to exploit cognitive vulnerabilities and reinforce engagement, making mobile gaming experiences increasingly comparable to other addictive behaviors (Haberlin & Atkin, 2022). Notably, the preference for mobile gaming and gaming on a computer may not stem from superior performance or usability. For example, Adepu and Adler (2016) found that although participants performed better on a word game when using a desktop computer, they still preferred playing on smartphones, citing convenience and ease of use as determining factors.

Elevated delay discounting rates, indicative of a preference for smaller immediate rewards over larger delayed ones, have been consistently associated with the severity of substance use and gambling disorders (Amlung, Vedelago, Acker, Balodis, & MacKillop, 2017; Bickel & Marsch, 2001). In the context of gaming, Buono et al. (2017) found that increased gaming duration was associated with higher delay discounting rates, irrespective of whether the reward involved monetary incentives or additional game time. Similarly, Weinstein, Abu, Timor, and Mama (2016) reported that individuals with gaming disorder not only tended to favor short-term rewards but also displayed heightened risk-taking behaviors, which can negatively impact academic and occupational functioning.

A study by Yan, Chen, Liu, and Zheng (2021) indicated that young adults with IGD exhibit impairments in response inhibition and reward-based decision-making, patterns comparable to those observed in individuals with nicotine dependence. A meta-analysis by Weinsztok, Brassard, Balodis, Martin, and Amlung (2021) comprising 78 studies (including 15 on gaming) demonstrated a medium to large effect size (Cohen's *d* = 0.53) in the association between behavioral addiction and delay discounting, even after removing a statistical outlier. Together, these studies suggest that steep delay discounting is a robust cognitive marker across both substance-related and behavioral addictions, including problematic gaming.

Despite growing concerns over mobile gaming, little research has directly examined the relationship between problematic mobile game use (PMGU) and delay discounting. Most existing studies have focused more broadly on general mobile phone use or mobile phone addiction. For example, Wilmer and Chein (2016) found that higher self-reported mobile device use was associated with steeper delay discounting and lower impulse control among university students. Similarly, Pancani, Petilli, Riva, and Rusconi (2023) reported that impulse control partially mediated the association between mobile phone use and delay discounting, suggesting a cognitive mechanism underlying these behaviors.

Moving beyond self-report, Schulz van Endert and Mohr (2020) conducted an objective assessment of smartphone use via the *iPhone's* built-in tracking system and categorized app use into 11 types. They found that the time spent on social and gaming apps was positively associated with delay discounting and negatively associated with impulse control. Using a novel task involving mobile phone battery power as a reward. Pancani et al. (2023) further demonstrated that individuals who scored higher on measures of smartphone-mediated communication (e.g., preference for digital over face-to-face interaction) also showed higher delay discounting tendencies. Notably, greater awareness of smartphone-related negative impacts was associated with lower delay discounting. These findings suggest that the amount and the type of mobile phone use, particularly in gaming, may influence individuals' intertemporal decision-making. However, the relationship between PMGU and delay discounting remains underexplored, highlighting a critical gap addressed by the present study.

Impulsivity has been identified as a key psychological factor associated with PMGU and excessive smartphone use (Chen et al., 2018; Jiang & Zhao, 2016; Schulz van Endert & Mohr, 2020; Shen, Zheng, & Zhang, 2023; West et al., 2021). Tang, Zhang, Yan, and Qu (2017) reported that individuals at high or moderate risk for mobile gaming addiction scored significantly higher on the 11-item Barratt Impulsiveness Scale (BIS-11) and showed a greater tendency to choose immediate rewards. Similarly, Robayo-Pinzon, Foxall, Montoya-Restrepo, and Rojas-Berrio (2021) tracked college students' mobile phone use over four weeks, and found that total screen time and time spent on specific apps such as *WhatsApp* and *Facebook* were significantly associated with mobile phone addiction. Moreover, addiction severity predicted performance on delay discounting tasks. Wilmer and Chein (2016) further demonstrated that mobile device use was positively associated with both delay discounting and reduced impulse control, with impulse control partially mediating this relationship. While time perception has received little attention in mobile gaming research, numerous studies have documented its distortion among individuals with elevated impulsivity. For example, those with ADHD (Walg, Hapfelmeier, El-Wahsch, & Prior, 2017; Zheng, Cheng, Sonuga-Barke, & Shum, 2022), individuals with orbitofrontal cortex damage (Berlin, Rolls, & Kischka, 2004), and high-impulsivity individuals (Barratt, 1983; Tsai & Yeh, 2014) have all been reported to associate with representing shorter estimation of time. A review by Moreira, Pinto, Almeida, and Barbosa (2016) concluded that individuals with impulsivity-related disorders frequently overestimate the passage of time and exhibit shorter time production, suggesting impaired temporal processing as a characteristic feature of impulsivity.

In summary, PMGU may share similar impulsive and compulsive mechanisms with other behavioral addictions. High impulsivity has been associated with steeper delay discounting and faster subjective time perception, which may lead individuals to overestimate waiting durations and devalue delayed rewards in favor of immediate gratification. Time perception, defined as an individual's subjective sense of elapsed time, has been proposed as a cognitive factor contributing to delay discounting (Jiang & Dai, 2021; Xu, González-Vallejo, & Vincent, 2020). According to the attention-gate model (Block & Zakay, 1996a), time perception is influenced by two primary factors: the internal pacemaker speed (affected by physiological arousal and cognitive stimulation) and the amount of attentional resources allocated to timing.

Recent studies suggest that a faster internal pacemaker increases delay discounting tendencies. For instance, Lee, Kable, and Jung (2024) found that shifting from fast to slow counting decreased discounting rates, whereas shifting from slow to fast increased them. Neuroimaging studies have further indicated that activity in brain regions such as the anterior insula and dorsomedial prefrontal cortex is modulated by timing speed. These findings support the idea that a faster internal clock may exaggerate the perceived cost of waiting and promote impulsive decisions.

On the other hand, studies in gaming contexts often report time underestimation. For example, experienced gamers tend to underestimate play duration compared to novices (Rau, Peng, & Yang, 2006), and adolescents underestimate gaming time more than reading time (Tobin & Grondin, 2009). This paradox may be explained by attentional absorption and distraction during gaming, especially among individuals with high impulsivity or attentional biases toward addiction cues (Wittmann & Paulus, 2008).

In an attempt to clarify these mixed findings, the present study investigated how impulsivity and time perception contribute to the association between problematic mobile gaming and delay discounting. Using time production tasks at multiple durations (60, 90, 120 s), the study aimed to capture both baseline and context-sensitive aspects of time perception and investigate the impact of impulsivity and time perception between problematic mobile gaming and delay discounting rate. Based on the extant literature, it was hypothesized that: (i) problematic mobile gaming would be positively associated with higher impulsivity and altered time perception (H_1_); (ii) altered time perception would be associated with higher impulsivity (H_2_); (iii) higher impulsivity would be associated with greater delay discounting (H_3_), and (iv) impulsivity and time perception would jointly mediate the relationship between problematic mobile gaming and delay discounting tendencies (H_4_).

## Methods

### Participants

A total of 98 participants (aged 18–45 years) were recruited through multiple online platforms, including *Facebook*, as well as flyers distributed through university networks. Those who self-reported with significant mental health illnesses, personality disorders, or other physiological conditions that may affect cognitive function were excluded. The participants were then invited to the laboratory for the experimental tasks. A power analysis conducted with G*power 3.133 indicated that at least 66 participants were needed to achieve statistical significance in a mixed-model ANOVA, assuming a medium effect size (*d* = 0.5), and a power of 0.8. Participants were required to record their mobile use or gaming time (hours per week), complete several psychometric scales, and participate in experimental delay discounting tasks. After completion of the study, each participant received NT$150 (New Taiwan Dollars; 30 NTD ≈ 1 USD) for their participation.

### Measures

#### Problematic Mobile Gaming Questionnaire (PMGQ)

The 12-item Problematic Mobile Gaming Questionnaire (PMGQ) (Pan et al., 2019) was used to assess problematic mobile gaming. Items (e.g., *“I think about playing mobile games even when I'm not playing”*) are rated on a four-point Likert scale from 1 (*strongly agree*) to 4 (*strongly disagree*) and assess three dimensions: compulsion, withdrawal, and tolerance. The scale exhibited excellent internal consistency in the present study (Cronbach's *α* = 0.92), with higher scores indicating more severe problematic mobile gaming. A cutoff score of 29/30 is recommended as the optimal threshold for differentiating between problematic mobile gamers and healthy individuals (Pan et al., 2019).

#### Barratt Impulsiveness Scale-11 (BIS-11)

The 30-item Barratt Impulsiveness Scale version 11 (BIS-11) (Patton, Stanford, & Barratt, 1995) was used to assess impulsivity. Items (e.g., “*I act on the spur of the moment*”) are rated on a four-point Likert scale from 1 (*rarely/never*) to 4 (*almost always/always*). The scale has three subscales: attentional impulsiveness (BIS_AI_), motor impulsiveness (BIS_MI_), and non-planning impulsiveness (BIS_NPI_) (Patton et al., 1995). The scale exhibited good internal consistency in the present study (Cronbach's *α* = 0.84), with higher scores indicating higher levels of impulsivity.

#### Time perception tasks

**Fig. 1.**
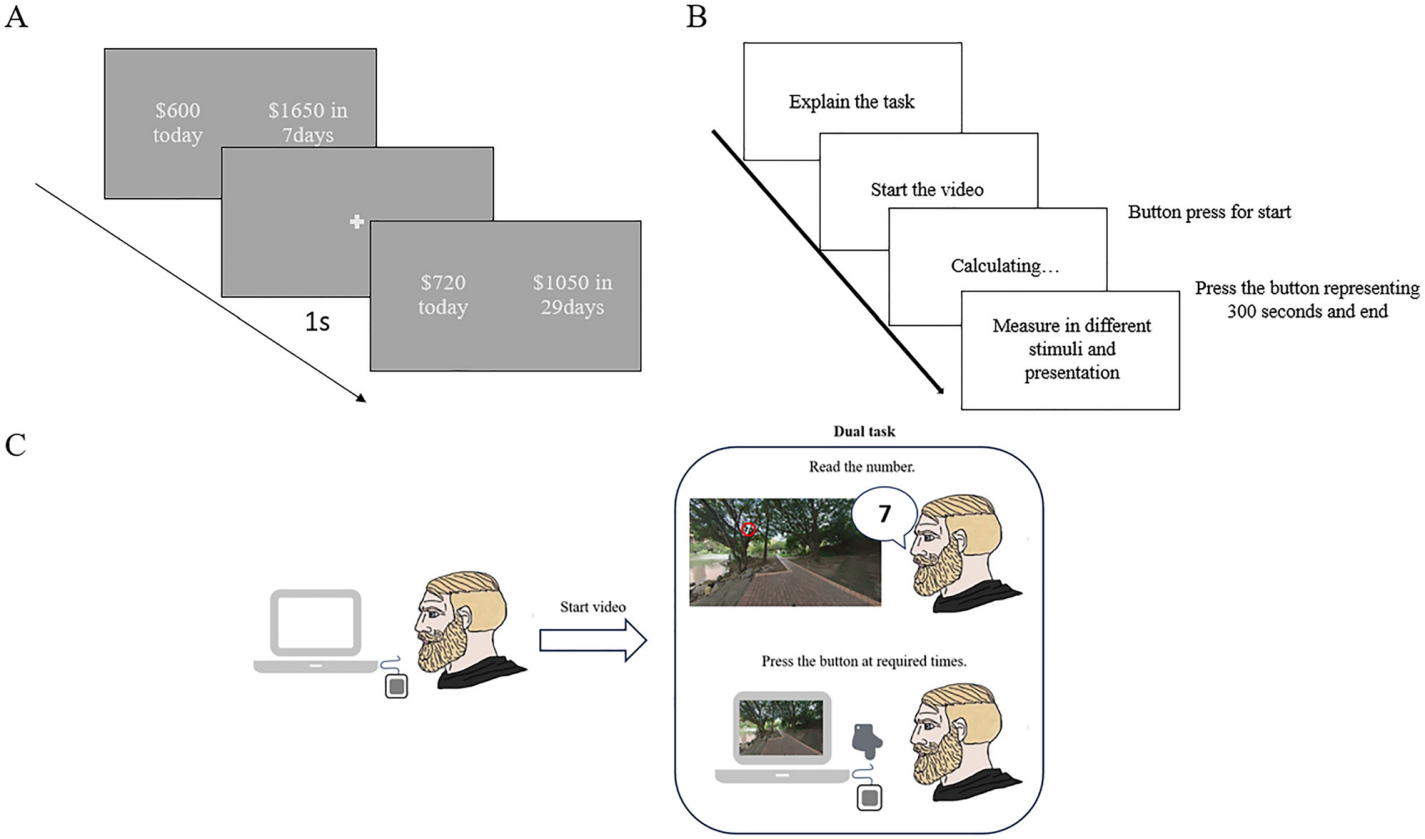
The experimental procedure of the tasks used in the present study. (A) Delay discounting task; (B) Time production task; (C) Concurrent distracter procedure in time production task

A time production task was used to assess how participants translated objective time into subjective time experiences for the time perception measurement. The procedure is shown in Fig. 1B. Participants were presented with a video clip (lasting 60, 90, 120 and 300 s) and were required to respond when they decided the time was up. Time intervals of 60, 90, 120, and 300 s were selected to capture variations in temporal estimation across short to extended durations, aligning with prior gaming-related studies (Rau et al., 2006; Tobin & Grondin, 2009; Wood & Griffiths, 2007). These durations provided an optimal balance between ecological validity and cognitive feasibility, avoiding the limitations of overly brief intervals that fail to reflect gaming immersion or excessively long ones that may induce fatigue and attentional drift. Within the task monitor, a number was embedded into the video as a distractor to avoid using mental calculation during the time production task (Fig. 1C).

The performance of time perception was based on relative error rate (Rer), which was calculated as the [subjective time (sT) − required time (rT)] / required Time (rT). Equation (1) below indicates that a value greater than 0 indicates an overproduction of time, implying that subjective time estimation was shorter and faster than the actual time, while a value less than 0 indicates an underproduction of time estimation.

(1) $$Rer=\frac{sT-rT}{rT}$$

#### Delay discounting task

As illustrated in Fig. 1A, participants chose between different sums of money available immediately or after a delay, with a one-second pause following each selection before the next question appeared. The task was computerized using *PsychoPy 2023.1.3* (https://www.psychopy.org), allowing participants to indicate their preferences using a mouse. The Extended Monetary Choice Questionnaire (MCQ-36), developed by Towe, Hobkirk, Ye, and Meade (2015) was used to measure delay discounting rates (DDRs). Each choice in the MCQ presents a trade-off between a smaller immediate reward and a larger delayed reward (e.g., *“$54 today or $80 in 30 days”*). The subjective value of delayed rewards is modeled using Mazur's hyperbolic discounting equation (2). The extended MCQ-36, designed to address ceiling effects in highly impulsive individuals, added nine items with higher *k* values (up to 4.0), expanding the rank range from 10 to 13. The delay discounting rate (DDR) was operationalized using the hyperbolic discount parameter *k*, which quantifies the degree to which individuals devalue rewards as a function of delay (Mazur, 1984; Towe et al., 2015).

(2) $$v_{immediate}=\frac{v_{\mathrm{d}elayed}}{1+kD}$$

Note: V_delayed_ is the delayed reward amount; *D* is the delay in days; *k* is the discounting rate parameter.

### Statistical analysis

Chi-square tests and ANOVAs were conducted to compare the differences between problematic mobile gamers (PMGs) and non-problematic mobile gamers (NPMGs) on the nominal variables and assessment scales. All analyses were conducted using *IBM SPSS version 25.0*. The effect size was calculated using Cohen's *d* (0.2 small, 0.5 medium, 0.8 large) (Cohen, 1988) and eta squared (*η*^2^) (0.01 small, 0.6 medium, and 0.14 large) (Richardson, 2011). Pearson's *r* correlation analyses were conducted to explore the associations between the variables. In addition, the mediating role of time perception and impulsivity in the relationship between problematic mobile gaming and delay discounting was examined using the *PROCESS macro for SPSS version 4.2* (Hayes, 2022). More specifically, Model 4 was employed to test a parallel mediation model, with time perception and impulsivity entered as simultaneous mediators. Indirect effects were estimated using a nonparametric bootstrap procedure with 5,000 resamples to calculate bias-corrected 95% confidence intervals.

### Ethics

The study was approved by the Institutional Review Board (IRB) at Tsaotun Psychiatric Center, Ministry of Health and Welfare (No. 111014). Before the study began, every participant was required to sign a consent form.

## Results

A total of 98 participants were recruited, and among the participants, 21 were classified as PMGs (21.4%) and 77 as NPMGs (78.6%) based on the PMGQ cutoff score (29/30). Among them, 39 were male (39.8%) and 59 were female (60.2%). The majority reported their age between 20 and 29 years (79.6%), and the distribution for other age groups was as follows: 15–19 years (16.3%), 20–29 years (80.6%), 30–39 years (2.0%), and over 40 years (1.0%). The PMGs reported a significantly higher tendency of behavioral impulsivity, a lower rate of time perception, and worse performance on the DDR (Table 1).

**Table 1.** Demographic characteristics of participants (*N* = 98)

Characteristics	nPMGs (*n* = 77)	PMGs (*n* = 21)	χ^2^/*Z/F* (*p*)
Gender *n* (%)			5.45 (.02)
Male	26 (33.8%)	13 (61.9%)	
Female	51 (66.2%)	8 (38.1%)	
Age group			.94 (.82)
15–19 years	12 (15.6%)	4 (19.1%)	
20–29 years	62 (80.5%)	17 (80.1%)	
30–40 years	2 (2.0%)	0 (0)	
Over 40 years	1 (1.0%)	0 (0)	
Frequency of mobile gaming (hours/week)			
Genre			6.14 (.29)
SPG	33 (42.86%)	9 (42.86%)	
FPS	7 (9.10%)	3 (14.29%)	
MMO	10 (12.99%)	1 (4.76%)	
MOBA	12 (15.58%)	7 (33.33%)	
RPG	8 (10.39%)	0 (0)	
Others	4 (5.19%)	1 (4.76%)	
Gaming experience			1.57 (.82)
≤1 year	8 (10.39%)	2 (9.52%)	
<3 years	4 (5.19%)	2 (9.52%)	
<5 years	10 (12.99%)	1 (4.76%)	
<7 years	10 (12.99%)	3 (14.29%)	
≥7 years	45 (58.44%)	13 (6.19%)	
PMGQ (M ± SD)	20.30 (5.47)	34.10 (3.59)	118.99 (<.00001)
BIS-11	54.31 (8.67)	61.14 (6.27)	11.38 (.001)
DDR (k)	−1.78 (.94)	−1.04 (1.11)	15.34 (<.0001)
DDR (Lnk)	.099 (.21)	.57 (.99)	9.46 (.003)
Subjective time passage			
60 s	61.25 (8.81)	73.72 (8.07)	34.18 (<.0001)
90 s	94.78 (13.48)	110.08 (14.69)	20.45 (<.0001)
120 s	127.98 (19.43)	148.83 (19.72)	18.88 (<.0001)
300 s	322.12 (58.35)	328.62 (64.22)	.20 (.66)
Relative error rate of time passage			
Rer_60–90_	.04 (.13)	.23 (.14)	31.67 (<.0001)
Rer_60–120_	.05 (.14)	.23 (.15)	29.53 (<.0001)
Rer_60–300_	.05 (.13)	.20 (.15)	18.16 (<.0001)

*Note*: PMGQ: Problematic Mobile Gaming Questionnaire; BIS: Barratt Impulsiveness Scale-11; DDR: Delayed discounting rate; Rer_60–90_: average relative error of time estimation of the 60 and 90 s; Rer_60–120_: the average relative error of time estimation of 60, 90, and 120 s; Rer_60–300_: the average relative error of time estimation of 60, 90, 120, and 300 s; SPG: Single Person Game; FPS: First Person Shooting; MMO: Massively Multiplayer Online Game; MOBA: Multiplayer Online Battle Arena; RPG: Role Play Game.

**Fig. 2.**
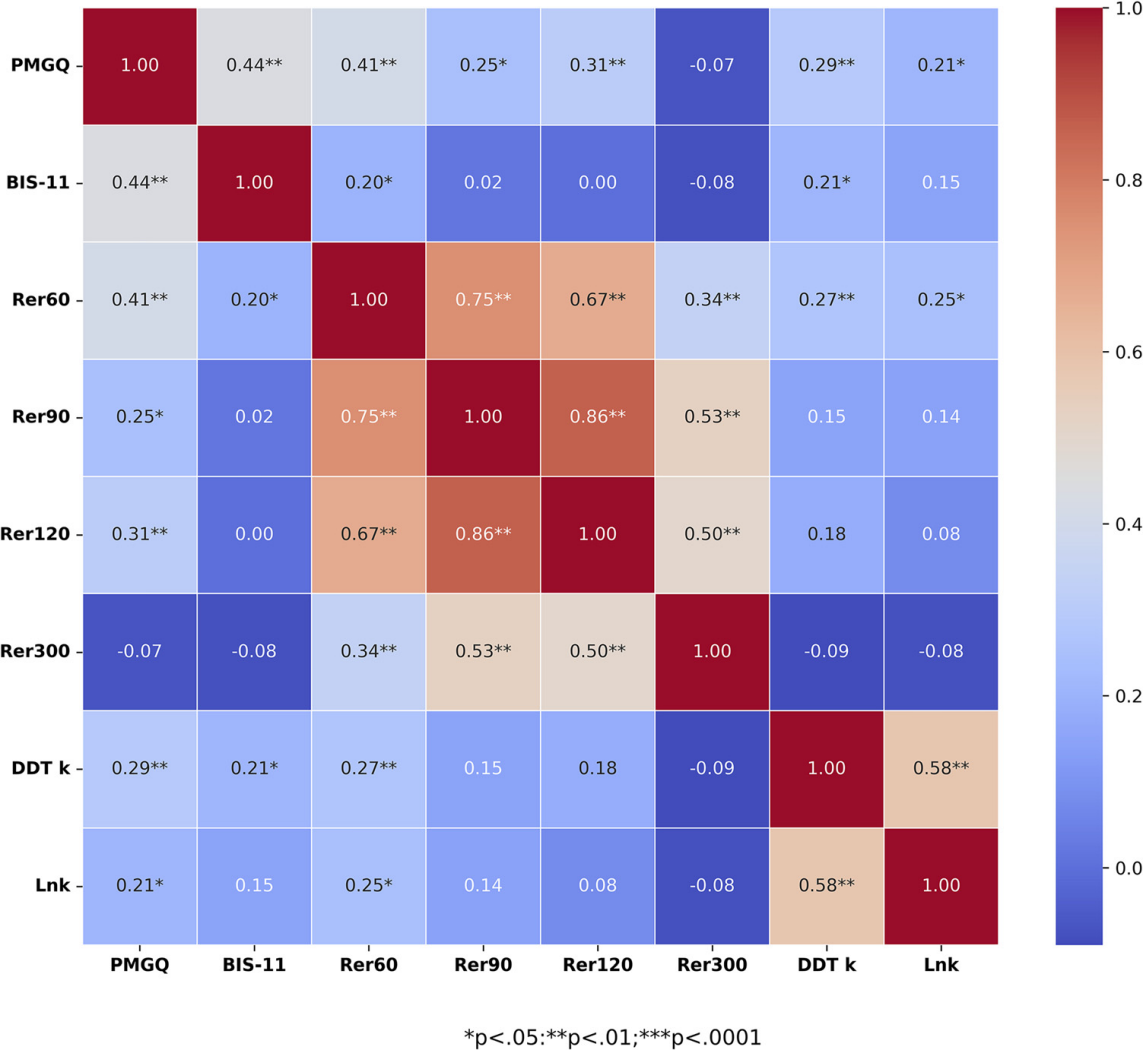
Relationship between PMGQ score, BIS-11 score, time perception, and delay discounting rate (lnk) *Note*: PMGQ: Problematic Mobile Gaming Questionnaire; BIS-11: Barratt Impulsiveness Scale, version 11.

In addition, correlation analyses showed significantly positive associations between problematic mobile gaming, impulsivity, relative error of time estimation of 60 (Rer_60_), and delay discounting rate, supporting H_1_ to H_3_ (Fig. 2). Moreover, taking subcomponents of BIS-11 to explore the correlation analysis, the results showed a significantly positive correlation between BIS subcomponents and PGMQ (r_AI-PMGQ_ = .36, *p* = .001*;* r_MI-PMGQ_ = .21, *p* = .04; r_NPI-PMGQ_ = .40, *p* < .0001, respectively), and between Rer_60_ and BIS_MI_ and BIS_NPI_ (r_MI-Rer60_ = .25, *p* =.02; r_AI-Rer60_ = .30, *p* = .004), respectively; but only between DDR and BIS_MI_ (r_MI-DDR_ = .30, *p* = .004).

Simple linear regression analyses examined whether problematic mobile gaming (PMGQ score) predicted trait impulsivity (BIS-11 total and subscale scores) and distorted time perception (Rer_60_). PMGQ scores significantly predicted overall impulsivity (*β* = 0.44, *t*[96] = 4.86, *p* < .001), explaining approximately 20% of the variance (*R*^2^ = .197, *F*[1,96] = 23.60, *p* < .001). This finding indicates that higher problematic gaming was associated with elevated impulsivity. Further analyses showed that PMGQ significantly predicted all BIS-11 subcomponents: BIS_AI_ (*β* = .36, *t* = 3.61, *p* = .001), BIS_MI_ (*β* = .21, *t* = 2.06, *p* = .043), and BIS_NPI_ (*β* = .40, *t* = 4.11, *p* < .001). PMGQ scores also significantly predicted distorted time perception (Rer_60_; *β* = 0.41, *t*[96] = 4.44, *p* < .001), accounting for 17% of the variance (*R*^2^ = .170, *F*[1,96] = 19.72, *p* < .001). Participants with higher PMGQ scores exhibited greater distortion in short-interval time estimation. Together, these results supported H_1_, indicating that problematic mobile gaming is associated with both increased impulsivity and altered time perception. A subsequent multiple regression analysis examined whether PMGQ, BIS-11, and Rer_60_ jointly predicted delay discounting rate (DDR). The overall model was significant (*F*[3,94] = 4.19, *p* = .008), explaining 11.8% of the variance (*R*^2^ = .118). Although PMGQ (*β* = 0.17, *t* = 1.49, *p* = .14) and BIS-11 (*β* = 0.09, *t* = 0.86, *p* = .394) were not individually significant predictors, the combination of predictors collectively contributed to explaining individual differences in delay discounting.

**Fig. 3.**
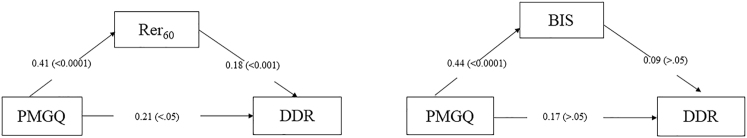
The time perception estimation indirectly mediated the association between problematic mobile gaming and delay discounting rate (DDR) *Note*: Rer_60_: relative error rate of time perception in 60 s; PMGQ: Problematic Mobile Gaming Questionnaire, DDR: Delay Discounting Rate, BIS-11: Barratt Impulsiveness Scale, version 11.

A parallel mediation analysis using PROCESS Model 4 (Hayes, 2022) tested whether impulsivity (BIS-11) and distorted time perception (Rer60) mediated the association between PMGQ and DDR. The overall model was significant (*F*[3,94] = 4.19, *p* = .008, *R*^2^ = .12). Neither BIS (*β* = 0.09, *p* = .39) nor PMGQ (*β* = 0.17, *p* = .14) independently predicted DDR, while Rer_60_ showed a trend-level association (*β* = 0.18, *p* = .093). The total effect of PMGQ on DDR was significant (*β* = 0.29, *p* = .004) but attenuated to non-significance when mediators were included (*β* = 0.17, *p* = .14), suggesting partial mediation. The indirect effect via BIS was not significant (Effect = 0.0028, 95% CI [−0.0013, 0.0070]), whereas the indirect effect via Rer_60_ was significant (Effect = 0.0051, 95% CI [0.0004, 0.0108]). Therefore, distorted time perception, but not impulsivity, significantly mediated the association between problematic gaming and delay discounting (Fig. 3).

## Discussion

The present study demonstrated a significant positive association between problematic mobile gaming (PMGQ) and trait impulsivity, consistent with H_1_ and prior research indicating that problematic mobile gaming is associated with elevated impulsivity traits such as poor self-regulation, urgency, and heightened reward sensitivity (Ioannidis et al., 2019; Kim et al., 2016). These findings suggest that individuals who engage in problematic gaming tend to make hasty decisions and exhibit diminished inhibitory control, reinforcing impulsive tendencies across digital and real-world contexts.

Moreover, impulsivity was positively associated with Rer_60_ and delay discounting (DDR) at the bivariate level, partially supporting H_2_ and H_3_, but it did not significantly predict DDR in the mediation model when controlling for PMGQ score. This partially contradicts H_4_, which posited that impulsivity would mediate the relationship between problematic mobile gaming and steep discounting of delayed rewards. One potential explanation is that the BIS-11 may not fully capture the specific temporal decision-making processes involved in intertemporal choice (Bickel, Koffarnus, Moody, & Wilson, 2014). It is also possible that state-based or task-specific impulsivity, rather than stable trait impulsivity, may play a more direct role in reward evaluation under delay. Multidimensional or behavioral measurements, such as the UPPS-P Impulsive Behavior Scale (Cyders & Smith, 2008) or Go/No-Go and Stop-Signal Tasks (Logan, Schachar, & Tannock, 1997; Aron, Fletcher, Bullmore, Sahakian, & Robbins, 2003), can be used to better assess the cognitive and affective aspects of impulsivity. Additionally, state impulsivity could be examined using ecological momentary assessment or physiological markers to capture situational fluctuations during gaming or reward-based tasks (Bickel et al., 2014).

In contrast, temporal processing distortions appear to be a more proximal mechanism potentially linking problematic gaming and delay discounting. PMGQ score had a significant direct effect on DDR even after controlling for impulsivity, while the indirect effect via impulsivity was non-significant. These findings align with Wood and Griffiths (2007), who found that time loss was frequently reported during video gameplay, particularly by female participants, but not necessarily associated with gaming severity. Instead, time loss was conceptualized as a byproduct of immersion and dissociation from external temporal cues. Similarly, in the present study, the observed mediation effect of time perception distortion at 60 s (Rer_60_) may reflect this dissociative state, whereby gamers who experience accelerated internal time perception may disproportionately favor immediate over delayed rewards.

More recently, Cervigón-Carrasco et al. (2023) extended this literature by experimentally demonstrating that subjective time passage (TP), as opposed to objective time estimation (TE), was more consistently associated with problematic gaming, pornography use, and binge-watching across diverse populations. Their findings showed that faster subjective time passage during media exposure predicted higher behavioral addiction scores across gaming, pornography, and binge-watching domains. These findings support those of the present study, suggesting that short-interval subjective time distortion may underlie impulsive choice behaviors among problematic mobile gamers by reducing temporal foresight and amplifying the subjective cost of delay.

Therefore, the present findings indicate that distorted time perception may serve as a core cognitive-affective mechanism linking gaming-related immersion with maladaptive intertemporal decision-making. While impulsivity remains a relevant personality risk factor, its non-significance in the present study's mediation model underscores the distinct contribution of online temporal experience in shaping decision-making. Individuals who consistently perceive time as passing more quickly may exhibit a compressed temporal window for planning and self-regulation (Bickel & Marsch, 2001), predisposing them to impulsive reward selection even in the absence of overt impulsivity.

Given that the study was conducted in Taiwan, cultural factors may have shaped the associations among mobile gaming, time perception, and impulsivity. In East Asian contexts, strong academic pressure, collectivistic values, and family expectations can foster technology-related addictive behaviors (Lee, Potenza, & Bhang, 2025). Within the I-PACE framework, affective (e.g., shame, fear of failure), cognitive (e.g., linking technology use with achievement), and executive (e.g., parental monitoring) influences may interact uniquely. These factors highlight the need to interpret findings within a cultural context and support culturally adapted applications of the I-PACE model (Brand et al., 2019; Zhang & Zeng, 2024). Importantly, among the mediators examined, subjective time perception at 60 s demonstrated the strongest and most consistent indirect effect on delay discounting. The present findings suggest that time perception at the 60-s interval served as the most salient mediator between problematic mobile gaming and impulsive decision-making. This temporal specificity may reflect the ecological characteristics of mobile gaming, which often involves short, repetitive, and reward-driven interactions. In such contexts, players make rapid decisions and receive immediate feedback, making shorter temporal intervals more cognitively salient and emotionally engaging (Luthman, Bliesener, & Staude-Müller, 2009; Rivero, Covre, Reyes, & Bueno, 2013). By contrast, longer intervals (e.g., 120–300 s) may be less representative of the real-world temporal structure of mobile gaming, where sustained attention and delayed feedback are uncommon. These results highlight the importance of considering the temporal scale of cognitive processing when examining impulsivity and reward sensitivity in mobile gaming behavior.

The selected 60–300 s intervals were designed to assess temporal perception within a range relevant to gaming while ensuring methodological rigor. Whereas prior studies used minute-level timing during active play (Rau et al., 2006; Tobin & Grondin, 2009; Wood & Griffiths, 2007), the present passive-viewing design required shorter intervals to capture attentional engagement without inducing fatigue, thereby maintaining ecological validity despite differing task demands.

This suggests that short-duration time perception is more cognitively salient and ecologically relevant to decision-making in everyday digital contexts. According to the attention-gate model (Zakay & Block, 1996), time perception is modulated by both pacemaker speed and attentional resource allocation. In high-arousal environments such as mobile gaming, attentional absorption may reduce temporal monitoring, leading to perceived acceleration or underestimation of time (Nuyens, Kuss, Lopez-Fernandez, & Griffiths, 2019; Tobin & Grondin, 2009). Such distortions may intensify the perceived aversiveness of delay, thereby promoting impulsive decisions. The absence of significant mediation effects at longer durations (e.g., 120 or 300 s) further suggests that shorter temporal intervals are more salient in behavioral outcomes such as mobile gaming and delay discounting. Contrary to expectations and some prior findings (Robayo-Pinzon et al., 2021; Yan et al., 2021), trait impulsivity, as assessed using the BIS-11, did not significantly mediate the relationship between problematic gaming and delay discounting. One explanation may be that impulsivity represents a broad personality disposition, while time perception distortion reflects a more domain-specific cognitive process directly implicated in decision-making under temporal constraints. Further analysis of BIS-11 subcomponents may clarify how impulsivity relates to temporal processing. Attentional impulsivity may distort timing by disrupting sustained focus, while non-planning impulsivity—reflecting neglect of future outcomes—is more directly linked to steeper delay discounting (Reynolds, Ortengren, Richards, & de Wit, 2006; Sharma, Markon, & Clark, 2014). In contrast, motor impulsivity shows weaker associations. These distinctions suggest that impulsivity facets influence time perception and intertemporal choice through distinct cognitive pathways in problematic mobile gaming.

Alternatively, the present study's modest sample size may have limited statistical power to detect smaller indirect effects. Future research should explore behavioral impulsivity tasks (e.g., Go/No-Go, delay discounting tasks) or subcomponents of impulsivity (e.g., non-planning vs. motor impulsivity) for a more nuanced understanding. Additionally, real-time experimental paradigms assessing the influence of in-game cues and reward immediacy on time perception and decision behavior could offer greater ecological validity.

Although the present study employed controlled laboratory tasks, the observed relationships between time perception, impulsivity, and gaming tendencies likely extend to real-world gaming environments. Game design elements such as countdowns, reward animations, and rapid feedback cycles are known to heighten attentional focus and reward anticipation, which may distort subjective time perception and encourage impulsive decisions (King, Delfabbro, & Griffiths, 2010; Wood & Griffiths, 2007). These features create dense temporal feedback loops that reinforce short-term reward seeking and reduce awareness of elapsed time. Therefore, the cognitive and affective mechanisms identified in the experimental tasks may mirror those embedded within actual gaming contexts, underscoring the ecological relevance of the present study's findings.

Finally, the present study's findings contribute to the literature on behavioral addiction and temporal decision-making. While impulsivity is often emphasized as a core feature of addictive behavior, the present study highlights the additional role of time perception, which may serve as a cognitive distortion pathway linking gaming disorder to maladaptive reward evaluation. In practical terms, interventions for problematic mobile gaming may benefit from targeting impulse control and cognitive training related to time estimation and future-oriented thinking (Snider, LaConte, & Bickel, 2016). Behavioral interventions such as mindfulness-based attention to time or episodic future thinking (EFT) may hold promise in reducing delay discounting biases. Mindfulness and EFT may also serve as an intervention for problematic mobile gaming, particularly when delivered through app-based or gamified formats. Brief mindfulness exercises or real-time prompts could help players regulate impulses during gameplay, while EFT tasks might encourage reflection on long-term goals and outcomes. Integrating these approaches within familiar digital environments may enhance engagement and target the cognitive and affective processes underlying impulsivity and temporal distortion in mobile gaming.

### Limitations

There are some limitations in the present study. Other potentially relevant mediators beyond time perception (Rer_60_) and impulsivity (BIS) may have contributed to the association between problematic mobile gaming and delay discounting. While impulsivity assessed using BIS-11 was included in a parallel mediation model, the non-significant mediating effect suggests measurement limitations or a more distal role in impulsive decision-making than time perception. In addition, the present study did not assess other potential confounders, such as sleep quality, stress levels, or comorbid behavioral addictions, which may also influence time perception and delay discounting. Future research should account for these factors to better isolate the effects of mobile gaming. Moreover, the reliance on a cross-sectional design precludes causal inferences about the directionality of effects among mobile gaming, cognitive mechanisms, and decision-making outcomes. Self-reported measures may also be subject to bias, such as underreporting of problematic behaviors or overestimation of cognitive control. Future studies employing longitudinal or experimental designs, along with neurocognitive or physiological indices, would enable more complex analyses to further clarify individual differences in the mechanisms linking mobile gaming, time perception, and impulsivity. Specifically, examining potential moderation effects by gender, game genre, or gaming intensity could provide valuable insights into subgroup variations in vulnerability and behavioral patterns. Longitudinal or experimental designs are necessary to elucidate the temporal dynamics and causal pathways between gaming behavior, cognitive distortions, and impulsivity, and to determine whether these cognitive processes act as antecedents or consequences of problematic mobile gaming.

## Conclusion

The present study provides novel evidence that subjective time perception distortion, particularly over short intervals, appears to play a key mediating role in the relationship between problematic mobile gaming and impulsive reward preference. While trait impulsivity was associated with both gaming behavior and delay discounting, it did not account for their relationship when modeled simultaneously. These findings underscore the importance of time perception as a core cognitive mechanism in the development of decision-making vulnerabilities associated with behavioral addictions. Interventions that enhance temporal monitoring and attentional regulation may prove especially valuable for individuals with problematic mobile gaming tendencies. Future studies should examine how dynamic environmental cues, attentional absorption, and emotional arousal influence timing and impulsivity within ecologically valid gaming scenarios.
